# Cerebral blood flow and glucose metabolism in healthy volunteers measured using a high-resolution PET scanner

**DOI:** 10.1186/2191-219X-2-63

**Published:** 2012-11-20

**Authors:** Marc C Huisman, Larissa W van Golen, Nikie J Hoetjes, Henri N Greuter, Patrick Schober, Richard G Ijzerman, Michaela Diamant, Adriaan A Lammertsma

**Affiliations:** 1 Department of Nuclear Medicine & PET Research, VU University Medical Center, Amsterdam, 1081, HV, The Netherlands; 2 Diabetes Center/Department of Internal Medicine, VU University Medical Center, Amsterdam, 1081, HV, The Netherlands; 3 Department of Anesthesiology, VU University Medical Center, Amsterdam, 1081, HV, The Netherlands

**Keywords:** Cerebral blood flow, Cerebral metabolic rate of glucose consumption, [^18^ F]FDG, Full kinetic analysis, [^15^O]H_2_O, High-resolution research tomograph, Image-derived input function, Parametric images

## Abstract

**Background:**

Positron emission tomography (PET) allows for the measurement of cerebral blood flow (CBF; based on [^15^O]H_2_O) and cerebral metabolic rate of glucose utilization (CMR_glu_; based on [^18^ F]-2-fluoro-2-deoxy-d-glucose ([^18^ F]FDG)). By using kinetic modeling, quantitative CBF and CMR_glu_ values can be obtained. However, hardware limitations led to the development of semiquantitive calculation schemes which are still widely used. In this paper, the analysis of CMR_glu_ and CBF scans, acquired on a current state-of-the-art PET brain scanner, is presented. In particular, the correspondence between nonlinear as well as linearized methods for the determination of CBF and CMR_glu_ is investigated. As a further step towards widespread clinical applicability, the use of an image-derived input function (IDIF) is investigated.

**Methods:**

Thirteen healthy male volunteers were included in this study. Each subject had one scanning session in the fasting state, consisting of a dynamic [^15^O]H_2_O scan and a dynamic [^18^ F]FDG PET scan, acquired at a high-resolution research tomograph. Time-activity curves (TACs) were generated for automatically delineated and for manually drawn gray matter (GM) and white matter regions. Input functions were derived using on-line arterial blood sampling (blood sampler derived input function (BSIF)). Additionally, the possibility of using carotid artery IDIFs was investigated. Data were analyzed using nonlinear regression (NLR) of regional TACs and parametric methods.

**Results:**

After quality control, 9 CMR_glu_ and 11 CBF scans were available for analysis. Average GM CMR_glu_ values were 0.33 ± 0.04 μmol/cm^3^ per minute, and average CBF values were 0.43 ± 0.09 mL/cm^3^ per minute. Good correlation between NLR and parametric CMR_glu_ measurements was obtained as well as between NLR and parametric CBF values. For CMR_glu_ Patlak linearization, BSIF and IDIF derived results were similar. The use of an IDIF, however, did not provide reliable CBF estimates.

**Conclusion:**

Nonlinear regression analysis, allowing for the derivation of regional CBF and CMR_glu_ values, can be applied to data acquired with high-spatial resolution current state-of-the-art PET brain scanners. Linearized models, applied to the voxel level, resulted in comparable values. CMR_glu_ measurements do not require invasive arterial sampling to define the input function.

**Trial registration:**

ClinicalTrials.gov NCT00626080

## Background

Positron emission tomography (PET) measurements using ^18^ F]-2-fluoro-2-deoxy-d-glucose (^18^ F]FDG) allow for quantitative determination of the cerebral metabolic rate of glucose utilization (CMR_glu_) [1,2]. Although, initially, rate constants were measured [3-5] using the compartment model developed by Sokoloff and co-workers [6], thereafter, CMR_glu_ measurements were increasingly based on the assumption of fixed rate constants in combination with static scans [7]. As a result, no recent estimates of the rate constants obtained using a state-of-the-art high-resolution scanner are available for human studies (estimates have only been reported for mice; see [8]).

Quantification of regional cerebral blood flow (CBF) based on ^15^O]H_2_O and PET also started around the same time period as metabolic assessments, i.e., around 1980. Due to hardware limitations, scanners were too slow for fast dynamic scans, leading to steady-state [9] and autoradiographic protocols [10]. Dynamic scan protocols were not introduced until the late 1980s [11,12], but to date, relatively few methodological papers on fully dynamic implementations have been published. In a review by Silverman and Phelps, it was noted that CBF measurements showed more variability than CMR_glu_ measurements with standard deviations within investigations of around 10% to 20% and up to 100% between investigations [13].

The high-resolution research tomograph (HRRT; CTI/Siemens, Knoxville, TN, USA) is a dedicated brain scanner that combines high spatial resolution (approximately 3 mm) with good sensitivity [14]. Previously, it has been shown that the increased spatial resolution allows for measurements of CMR_glu_ with reduced underestimation due to partial volume effects [15,16]. These glucose measurements, however, were based on a static FDG protocol together with population average-based values for the rate constants. In a recent study, HRRT image quality was shown to be good enough to allow for quantitatively correct CBF measurements [17].

The use of an arterial line (blood sampler derived input function (BSIF) to acquire an input function is the gold standard for dynamic data analysis of cerebral studies, but it is an invasive procedure. Therefore, use of an image-derived input function (IDIF) can be an interesting alternative [18-20], but its utility needs to be validated for each tracer, each scanner, and each acquisition and data analysis protocol separately.

The main purpose of the present study was to derive human CBF and CMR_glu_ values as measured using a current state-of-the-art high-resolution scanner. Data were analyzed by full kinetic analysis using nonlinear regression (NLR) of regional time-activity curve (TAC) data with a BSIF. A second objective was to assess the accuracy of high-resolution parametric images of CBF and CMR_glu_. In addition, CBF and *K*_i_ values for automatically delineated regions were compared with manually drawn regions of interest and literature values. Based on earlier work on IDIFs [18,20,21] and given the high resolution of the HRRT, an additional objective was to assess whether a carotid artery-based IDIF could be used as a noninvasive alternative for arterial sampling in the case of both CBF and CMR_glu_ measurements, thereby increasing clinical applicability of this methodology in humans.

## Methods

### Healthy subjects and study design

Thirteen healthy men (age 36.2 ± 13.2 years, body mass index 25.8 ± 3.7 kg/m^2^, arterial plasma glucose 5.5 ± 0.2 mmol/L) participated in this study. The study consisted of a screening visit and two visits for magnetic resonance imaging (MRI) and PET scan acquisition, respectively. All subjects were free of medical and psychiatric illness based on medical history, physical examination, and blood analysis. All subjects provided written informed consent prior to inclusion. The study was approved by the Medical Ethics Review Committee of the VU University Medical Center and the Central Committee on Research Involving Human Subjects; the study was conducted according to the Declaration of Helsinki.

### Scan protocol

One week prior to the PET study, 3-D structural MRI images were acquired using a 3.0 T GE Signa HDxt MRI scanner (General Electric, Milwaukee, WI, USA) and a T1-weighted fast spoiled gradient echo sequence. Data consisted of 172 planes of 256 × 256 voxels with a voxel size of 0.94 × 0.94 × 1 mm^3^.

At the day of the PET study, catheters were placed in the antecubital vein for tracer injection and in the radial artery for blood sampling. Next, subjects were positioned on the HRRT scanner bed such that the head was in the center of the field of view. Velcro tapes were used to minimize patient movement during the entire imaging procedure. Lights were dimmed, noise was minimized, and subjects were asked to close their eyes and stay awake during data acquisition. Prior to or immediately after the ^15^O]H_2_O scan, a 6-min singles-based transmission scan with a fan-collimated ^137^Cs moving point source was acquired. A 10-min dynamic emission scan was started 10 s prior to a bolus injection of approximately 800 MBq ^15^O]H_2_O. Radioactivity was contained within a volume of approximately 5 mL and was followed by a saline flush to give a total injected volume of 40 mL, administered at a rate of 2 mL/s using an infusion pump (Medrad Inc., Indianola, MS, USA). To allow for radioactive decay of ^15^O, the ^18^ F]FDG scan was acquired after an interval of at least 10 min following the ^15^O]H_2_O scan. A 60-min dynamic emission scan was started 30 s prior to a bolus injection of 186 ± 9 MBq ^18^ F]FDG. The administration protocol was identical to the one used for the ^15^O]H_2_O scan. During both emission scans, arterial blood concentrations were monitored continuously using a dedicated on-line blood sampler (Veenstra Instruments, Joure, Netherlands [22]). During the first 4 min of the ^15^O]H_2_O scan, blood was withdrawn at a rate of 450 mL/h and activity was read out once per second. For the remaining 6 min, the withdrawal rate was reduced to 150 mL/h and the readout sampling time increased to 10 s. In addition, three manual blood samples were taken at 5, 7.5, and 9 min post-injection. These samples were taken from the same arterial line by briefly interrupting continuous withdrawal. After each sample, the arterial line was flushed with heparinized saline to prevent clotting. Manual samples were used to measure whole blood radioactivity concentrations. The sampling procedure during the ^18^ F]FDG scan was similar to that of the ^15^O]H_2_O scan but with withdrawal rates of 300 mL/h during the first 5 min and 150 mL/h thereafter. During this scan, manual blood samples were taken 15, 35, and 55 min post-injection. These samples were used to measure both whole blood and plasma radioactivity concentrations, as well as arterial plasma glucose levels. This BSIF was used in all analyses except for analyses based on an IDIF.

### Data reconstruction

Emission data were histogrammed into multiframe sinograms (^15^O]H_2_O 14 fames of 6 × 10, 2 × 30, 4 × 60, and 2 × 120 s; ^18^ F]FDG 19 frames of 6 × 10, 2 × 30, 3 × 60, 2 × 150, 2 × 300, and 4 × 600 s). Sinograms were normalized, corrected for randoms, dead time, and decay, and based on the transmission scan corrected for scatter and attenuation. Corrected sinograms were reconstructed using the iterative 3D ordinary Poisson ordered subset expectation maximization algorithm [23,24] using eight iterations and 16 subsets. Reconstructed images consisted of 207 planes of 256 × 256 voxels with a voxel size of 1.22 × 1.22 × 1.22 mm^3^.

### Regions of interest definition

The MRI image was co-registered with either a corresponding summed ^18^ F]FDG (15 to 60 min) or ^15^O]H_2_O (0 to 90 s) image using the software package VINCI [25]. Both PET and MRI images were rebinned and cropped into 128 × 128 × 63 matrices with an isotropic voxel dimension of 2.44 mm. Automatic delineation of regions of interest (ROIs) was performed using PVElab [26] resulting in a total of 17 gray matter regions, subdivided into their left and right constituents (cerebellar cortex, orbital frontal cortex, inferior medial frontal cortex, anterior cingulate cortex, thalamus, insula, caudate nucleus, putamen, superior temporal cortex, parietal cortex, inferior medial temporal cortex, superior frontal cortex, occipital cortex, sensory motor cortex, posterior cingulate cortex, enthorinal cortex, and hippocampus), a global white matter (WM) region, and a total gray matter (GM) region. To compare our data to literature values, two additional manually drawn ROIs were analyzed: a gray matter region (insular gray in four successive transversal planes) and a white matter region (centrum semiovale in two successive transversal planes), using Amide [27]. Corresponding TACs were generated by projecting these ROIs onto the dynamic image sequences.

### Calculation of CMR_glu_

Sampler data were corrected for flushes and calibrated using the plasma concentrations derived from the three manual samples per subject to generate an arterial plasma input function. First, using NLR, TACs were fitted to the standard irreversible two-tissue compartment model, providing the three rate constants *K*_1_, *k*_2_, and *k*_3_ as well as the blood volume fraction *V*_B_. Second, the validity of the Patlak linearization [28] was investigated by comparing regional values of the net influx rate constant *K*_i_ with those obtained using NLR. Third, the Patlak method was used (without smoothing) on a voxel-by-voxel basis, and for each ROI, mean values extracted from parametric *K*_i_ images were compared with those obtained from regional Patlak analyses. Additionally, parametric images were smoothed with a 6-mm Gaussian filter (typical resolution of the current-generation whole-body PET scanners) prior to analysis. CMR_glu_ was calculated as *K*_i_ times arterial plasma glucose divided by a lumped constant of 0.52 [29].

### Calculation of CBF

Whole blood input functions as well as regional TACs were derived as described above. First, TACs were fitted to the standard single-tissue compartment model, fixing delay and dispersion to the values obtained from a fit to the whole brain TAC [12], providing CBF as well as *V*_T_, distribution volume. Second, after smoothing with a 6-mm Gaussian filter, this analysis was repeated, and in addition, parametric CBF images were generated using a basis function method (BFM) implementation of the blood flow model [30]. For each ROI, mean parametric CBF was compared with the corresponding value from the regional analysis. The FDG extraction fraction was calculated from combined CBF and CMR_glu_ data as *K*_1_ = *E* · CBF.

### Image-derived input functions

BSIFs are invasive, and therefore, the use of IDIFs was investigated. For FDG, arterial activity was clearly seen after smoothing an early frame (approximately 20 to 30 s post-injection) with a 6-mm Gaussian filter. Starting three planes below the circle of Willis to avoid contamination with activity from the brain, ten successive planes were combined into an ROI representing a carotid artery [18,19]. The four hottest pixels per plane were identified and combined in a carotid artery ROI. The average time-activity curve obtained from the two carotid artery ROIs, scaled to the manual samples, was taken as the whole blood IDIF. A plasma IDIF was obtained by multiplying the whole blood IDIF with the average plasma-to-whole blood ratio derived from the samples. For ^15^O]H_2_O, an identical procedure was followed.

## Results and discussion

### Results

[^18^ F]FDG scans were acquired in all 13 subjects and [^15^O]H_2_O scans in 12 subjects (at one occasion, no [^15^O]H_2_O was available). Analysis of one [^18^ F]FDG scan failed due to sampling problems, and two [^18^ F]FDG scans were discarded because of movement artifacts. Due to technical problems, [^18^ F]FDG and [^15^O]H_2_O data could not be obtained for one subject. Overall, 9 complete [^18^ F]FDG and 11 complete [^15^O]H_2_O data sets were available for analysis, and 8 combined data sets could be used for the calculation of the FDG extraction fraction.

#### CMR_glu_

No significant differences between left and right CMR_glu_ values were observed for any of the regions delineated automatically. Average (left and right) CMR_glu_ values are listed in Table 1. Total gray matter CMR_glu_ was 0.29 ± 0.03 μmol/cm^3^ per minute, and white matter CMR_glu_ was 0.19 μmol/cm^3^ per minute (ratio GM/WM = 1.5). Excluding the entorhinal cortex, an average coefficient of variation (CoV) of 10.0% was observed. The high CoV in the entorhinal cortex is probably due to the small volume of this region (3.4 ± 0.4 mL), and this region was excluded from the further analyses. A typical blood volume fraction, *V*_B_, of 0.05 ± 0.01 was obtained.

**Table 1:** **Regional CMR**_**glu**_**, *K***_**1**_**, CBF, *V***_**T**_**, and *E *values obtained in healthy males**

**Region**	**CMR**_**glu**_	***K***_**1**_	**CBF**	***V***_**T**_	***E***
Cerebellum	0.24 ± 0.03 (11)	0.075 ± 0.011 (15)	0.36 ± 0.07 (19)	0.71 ± 0.08 (11)	21 ± 2 (11)
Orbitofrontal cortex	0.34 ± 0.04 (10)	0.065 ± 0.010 (15)	0.42 ± 0.07 (17)	0.69 ± 0.05 (7)	16 ± 2 (14)
Medial inferior frontal cortex	0.39 ± 0.03 (8)	0.073 ± 0.012 (16)	0.42 ± 0.06 (13)	0.71 ± 0.05 (7)	17 ± 3 (15)
Cingulate anterior	0.30 ± 0.04 (12)	0.065 ± 0.009 (14)	0.37 ± 0.05 (13)	0.69 ± 0.05 (8)	17 ± 3 (16)
Thalamus	0.36 ± 0.04 (11)	0.082 ± 0.018 (22)	0.46 ± 0.06 (13)	0.74 ± 0.05 (7)	19 ± 3 (16)
Insula	0.33 ± 0.03 (10)	0.069 ± 0.007 (11)	0.45 ± 0.07 (15)	0.76 ± 0.05 (7)	16 ± 2 (10)
Caudate nucleus	0.38 ± 0.04 (11)	0.067 ± 0.011 (16)	0.39 ± 0.06 (16)	0.68 ± 0.07 (10)	18 ± 3 (17)
Putamen	0.41 ± 0.04 (10)	0.076 ± 0.013 (17)	0.47 ± 0.08 (16)	0.75 ± 0.07 (9)	17 ± 4 (21)
Superior temporal cortex	0.33 ± 0.03 (8)	0.064 ± 0.008 (13)	0.38 ± 0.04 (11)	0.70 ± 0.05 (7)	17 ± 2 (10)
Parietal cortex	0.37 ± 0.02 (6)	0.072 ± 0.009 (12)	0.39 ± 0.05 (12)	0.71 ± 0.05 (7)	19 ± 2 (11)
Medial inferior temporal cortex	0.31 ± 0.03 (11)	0.059 ± 0.007 (13)	0.33 ± 0.05 (15)	0.65 ± 0.06 (10)	18 ± 2 (11)
Superior frontal cortex	0.36 ± 0.03 (8)	0.069 ± 0.009 (13)	0.40 ± 0.06 (14)	0.71 ± 0.05 (7)	17 ± 2 (12)
Occipital cortex	0.35 ± 0.03 (7)	0.067 ± 0.007 (10)	0.38 ± 0.05 (14)	0.68 ± 0.07 (10)	18 ± 1 (8)
Sensorimotor cortex	0.34 ± 0.02 (6)	0.068 ± 0.009 (13)	0.37 ± 0.04 (12)	0.68 ± 0.05 (7)	19 ± 2 (12)
Cingulate posterior	0.41 ± 0.03 (8)	0.079 ± 0.013 (16)	0.44 ± 0.06 (13)	0.73 ± 0.05 (7)	19 ± 2 (12)
Enthorinale	0.19 ± 0.08 (44)	0.047 ± 0.014 (30)	0.26 ± 0.05 (18)	0.59 ± 0.08 (14)	18 ± 4 (25)
Hippocampus	0.22 ± 0.04 (16)	0.057 ± 0.011 (19)	0.30 ± 0.04 (14)	0.67 ± 0.07 (10)	19 ± 4 (20)
White matter	0.19 ± 0.01 (7)	0.046 ± 0.004 (9)	0.25 ± 0.04 (15)	0.61 ± 0.05 (9)	19 ± 2 (10)
Total gray matter	0.29 ± 0.03 (8)	0.062 ± 0.006 (10)	0.35 ± 0.05 (14)	0.64 ± 0.05 (8)	18 ± 1 (8)

Mean ± SD (CoV); data are obtained by NLR analysis based on BSIFs. CMR_glu_, cerebral metabolic rate of glucose consumption (in μmol/cm^3^ per minute (*n* = 9)); *K*_1_, rate constant from blood to tissue (in min^-1^ (*n* = 9)); CBF, cerebral blood flow (in mL/cm^3^ per minute (*n* = 11)); *V*_T_, volume of distribution (unitless, obtained by CBF measurements (*n* = 11)); *E*, FDG extraction fraction (in % (*n* = 8)).

Manually drawn ROIs resulted in CMR_glu_ values of 0.33 and 0.11 μmol/cm^3^ per minute for gray matter and white matter, respectively (ratio GM/WM = 3.0). In Table 2, mean values of the separate rate constants for these manual ROIs are listed as well as a comparison with literature data in which similar types of ROIs were used; no recent papers were available for comparison of these separate parameters.

**Table 2:** Rate constants of FDG parameters for manually drawn gray and white matter ROIs and literature values

	**FDG parameters**				
	***K***_**i**_	***K***_**1**_	***K***_**2**_	***k***_**3**_	***k***_**4**_
Present study (3 K)					
GM	0.031 ± 0.004	0.062 ± 0.008	0.071 ± 0.04	0.067 ± 0.03	n/a
WM	0.010 ± 0.0008	0.033 ± 0.004	0.083 ± 0.03	0.037 ± 0.01	n/a
GM/WM	3.0	1.9	0.87	1.8	n/a
Huang et al. (4 K) [3]					
GM	0.0334 ± 0.006	0.102 ± 0.03	0.13 ± 0.07	0.062 ± 0.02	0.0068 ± 0.001
WM	0.0154 ± 0.004	0.054 ± 0.01	0.109 ± 0.04	0.045 ± 0.02	0.0058 ± 0.002
GM/WM	2.2	1.9	1.2	1.4	1.2
Reivich et al. (3 K) [29]					
GM	0.035^a^	0.105 ± 0.006	0.148 ± 0.008	0.074 ± 0.005	n/a
WM	0.023^a^	0.069 ± 0.005	0.129 ± 0.004	0.064 ± 0.006	n/a
GM/WM	1.5	1.5	1.1	1.2	n/a
Reivich et al. (4 K) [29]					
GM	0.034^a^	0.095 ± 0.005	0.125 ± 0.002	0.069 ± 0.002	0.0055 ± 0.0003
WM	0.022^a^	0.065 ± 0.005	0.126 ± 0.003	0.066 ± 0.002	0.0054 ± 0.0006
GM/WM	1.5	1.5	0.99	1.0	1.0

Mean ± SD; data of the present study are obtained by NLR analyses based on BSIFs. 3 K, 3 *k* model (i.e., *k*_4_ = 0); 4 K, 4 *k* parameter model; *K*_i_, net influx rate (in min^-1^); *K*_1_, rate of transport from blood to brain (in min^-1^); *k*_2_, rate of transport from brain to blood (in min^-1^); *k*_3_, phosphorylation rate by hexokinase (in min^-1^); *k*_4_, rate of hydrolysis by glucose-6-phosphatase (in min^-1^); *GM*, gray matter; *WM*, white matter; n/a, not applicable. ^a^Calculated by the *K*_1_, *k*_2_, and *k*_3_ parameters reported in the paper.

**Figure 1:**
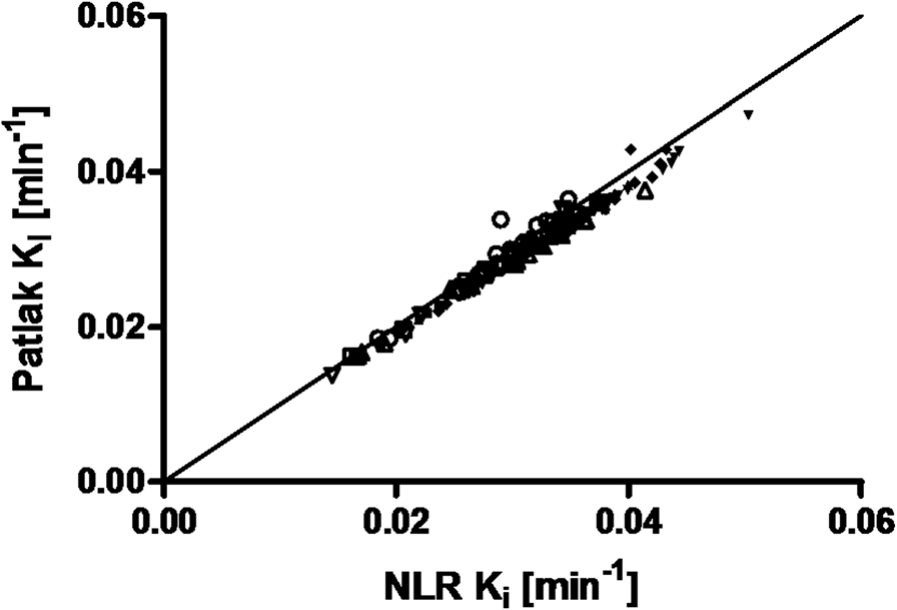
**Correlation between Patlak- and NLR-derived *K***_**i**_**values.** Data of all 16 gray matter regions and a white matter brain region of nine healthy subjects are presented. Data points for each individual subject are shown with a separate symbol. The solid line indicates the line of identity.

Figure 1 shows the (linear) relationship between Patlak- and NLR-derived *K*_i_ values for the 16 (automatic) combined gray matter regions and a white matter region. Linear regression provided a slope of 0.96 and an *r*^2^ of 0.98.

**Figure 2:**
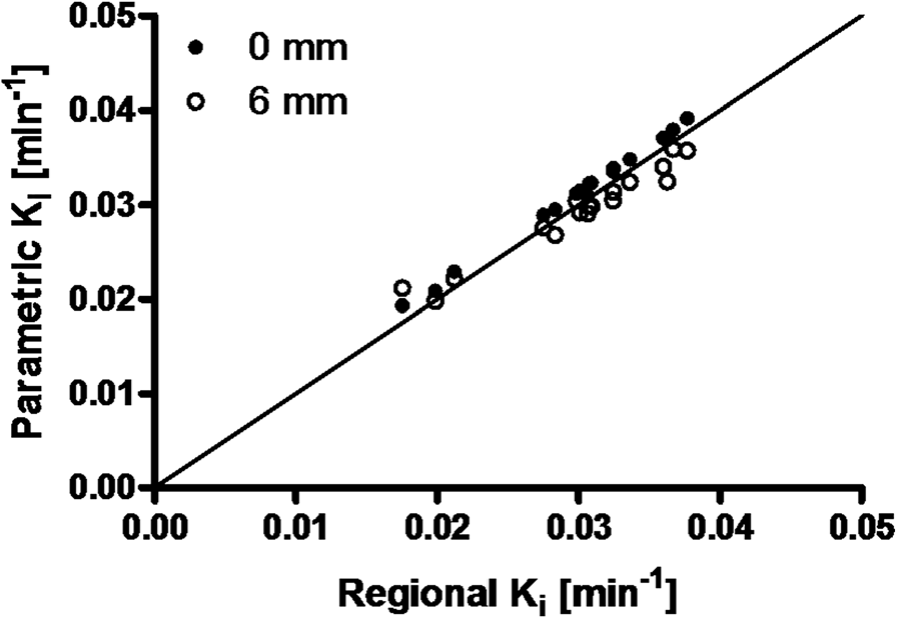
**Correlation of average *K***_**i**_**values derived using parametric and regional Patlak analyses.** Data of all 16 total gray matter regions and a white matter brain region are presented for nine subjects. Parametric values represent the mean of all voxels within an ROI. The solid line indicates the line of identity. Results for both images without smoothing (black dots) and those smoothed with a 6-mm Gaussian filter (white dots) are shown.

Based on the good correlation between Patlak and NLR results, parametric (Patlak) images were generated without smoothing. Figure 2 shows the relationship between average parametric and ROI-derived *K*_i_ values, both before and after smoothing of the parametric images. Without smoothing, a slope of 1.04 and an *r*^2^ of 0.99 were obtained. After smoothing, these values were 0.97 and 0.90, respectively.

**Figure 3:**
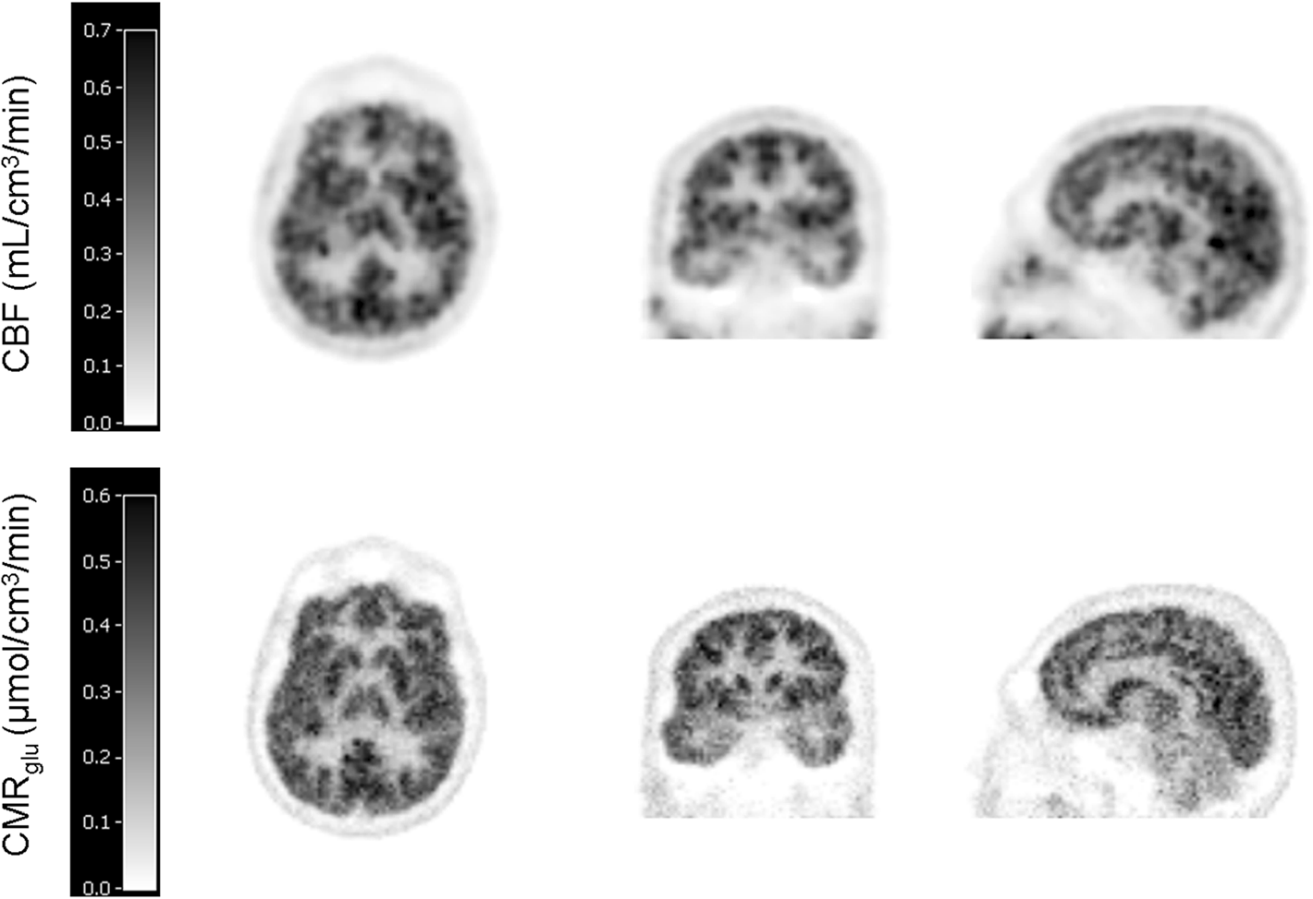
**Representative parametric images of a single subject.** The CBF image (upper panel) and the CMR_glu_ image (lower panel) of the same subject are presented. The parametric CBF image was generated after smoothing with a 6-mm Gaussian filter.

The correlation between average regional NLR and parametric values had a slope of 1.0 and *r*^2^ of 0.90 (data not shown). A representative parametric image is shown in Figure 3.

#### CBF

**Figure 4:**
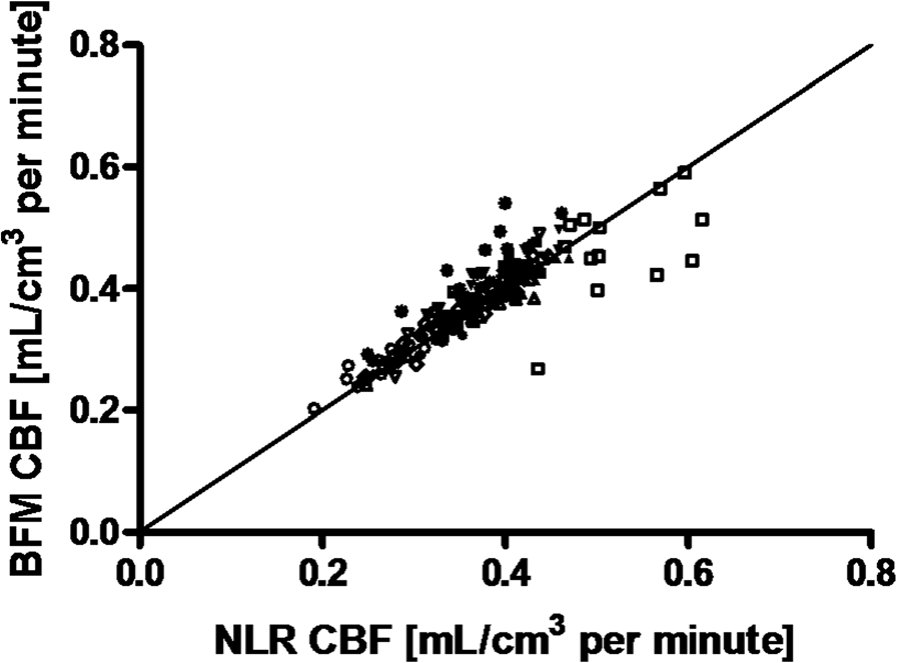
**Correlation of CBF values derived using parametric (basis function method) and regional (NLR) analyses.** Data of 16 gray matter regions and a white matter region are shown for 11 subjects. Parametric values represent the mean of all voxels within an ROI. Data points for each individual subject are shown with a separate symbol. The solid line indicates the line of identity.

Average delay and dispersion values were 12 ± 3 and 10 ± 2 s, respectively. CBF values for the various automatically delineated regions using NLR analyses are given in Table 1. Total gray and white matter CBFs were 0.35 ± 0.05 and 0.25 ± 0.04 mL/cm^3^ per minute, respectively, (GM:WM = 1.4). Table 3 shows CBF values obtained from the manually drawn gray and white matter regions (ratio GM/WM = 3.3) as well as a comparison with literature data, recently obtained using another HRRT scanner [17]. Figure 4 shows the relationship between CBF values derived from BFM and NLR analyses on smoothed data. Linear regression provided a slope of 1.02 and an *r*^2^ of 0.73. In Figure 3, a representative parametric image is presented.

**Table 3:** **Rate constants of H**_**2**_**O parameters for manually drawn gray and white matter ROIs and literature values**

	**H**_**2**_**O parameters**		
	**CBF**	***V***_**T**_	***E***
Present study			
GM	0.43 ± 0.09	0.72 ± 0.06	15
WM	0.13 ± 0.02	0.69 ± 0.10	26
GM/WM	3.3	1.1	0.6
Walker et al. [17]			
GM	0.44 ± 0.03		
WM	0.15 ± 0.03		
GM/WM	2.9		

Mean ± SD; data of the present study are obtained by NLR analyses based on BSIFs. *CBF*, cerebral blood flow (in mL/cm^3^ per minute); *V*_T_, volume of distribution (unitless); *E*, FDG extraction fraction (in %); *GM*, gray matter; *WM*, white matter.

FDG extraction fractions for each ROI are shown in Table 1. For the total gray matter region, the FDG extraction fraction was 18 ± 1%.

#### Image-derived input functions

**Figure 5:**
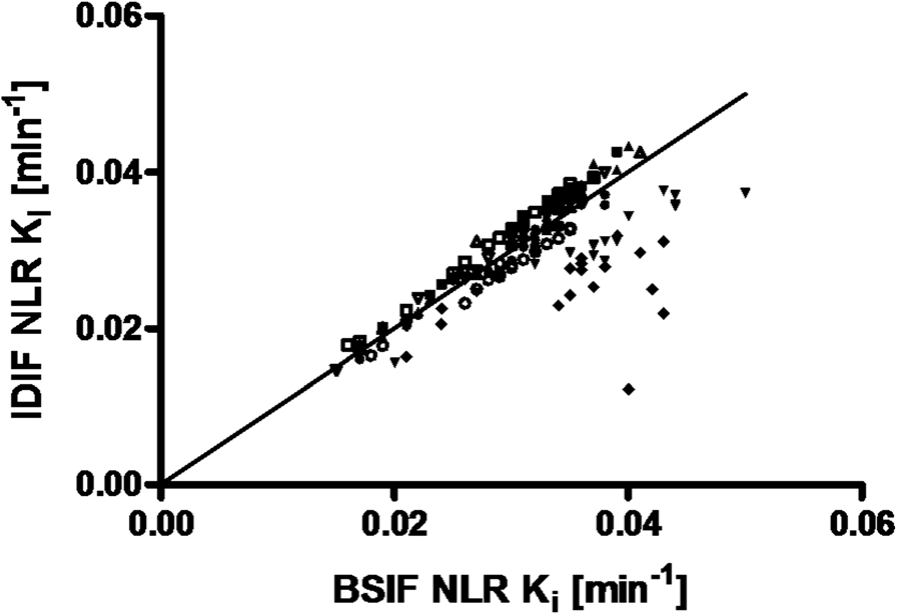
**Correlation between IDIF- and BSIF-based NLR-derived *K***_**i**_**values.** The correlation is for 16 gray matter regions and a white matter region. Data points for each individual subject (*n* = 9) are shown with the same symbol. The solid line indicates the line of identity.

In order to scale the IDIF derived from the [^18^ F]FDG scan to the manual samples, a multiplication factor was derived (average value 2.2 ± 0.5). Figure 5 shows the relationship between IDIF- and BSIF-derived *K*_i_ values for regional NLR ROI analyses. In two out of nine subjects, a slope of 0.76 ± 0.09 and *r*^2^ of 0.40 ± 0.65 were observed. For the other seven patients, slope and *r*^2^ were 1.03 ± 0.05 and 0.98 ± 0.01, respectively.

**Figure 6:**
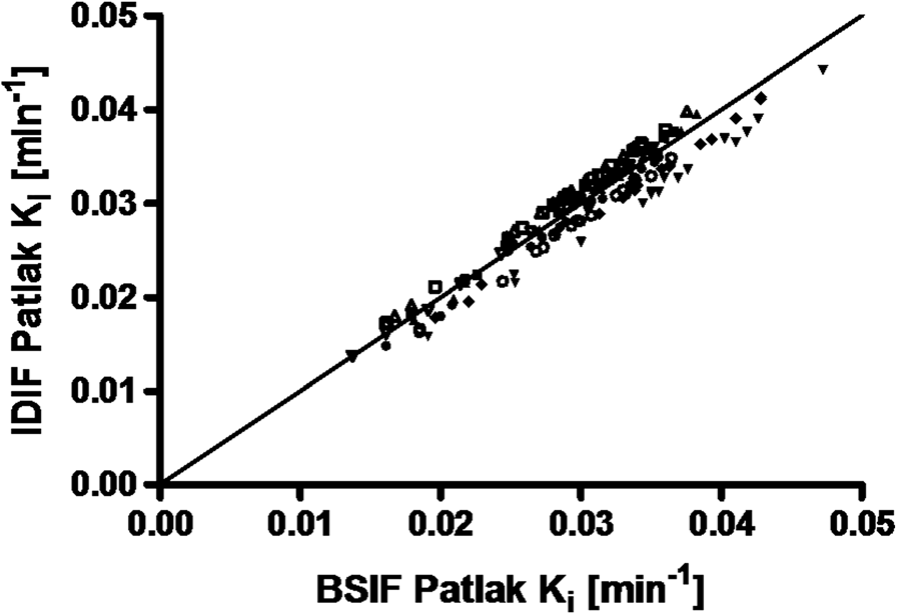
**Correlation between IDIF- and BSIF-based Patlak-derived *K***_**i**_**values.** The correlation is for 16 gray matter regions and a white matter region. Data points for each individual subject (*n* = 9) are shown with the same symbol. The solid line indicates the line of identity.

Figure 6 shows the results of a similar comparison for Patlak-derived *K*_i_ values. In this case, no apparent outliers, either in single fits or complete subjects, were apparent. Slope and *r*^2^ were 0.99 ± 0.06 and 0.99 ± 0.007, respectively.

In order to scale the IDIF derived from the [^15^O]H_2_O scan to the manual samples, a multiplication factor of 1.8 ± 0.4 (averaged over 11 subjects) was needed. However, after scaling, a BSIF/IDIF peak ratio of 3.0 ± 1.2 was observed. Therefore, the shape of the IDIF and the simultaneously measured BSIF did not match, and thus, an IDIF approach was not possible in this case.

## Discussion

To date, no full kinetic analysis of [^18^ F]FDG data based on arterial sampling and a dynamic HRRT scanning protocol in humans has been reported. In the present study, average CMR_glu_ values of 0.33 and 0.11 μmol/cm^3^ per minute were obtained for manually drawn gray and white matter regions, respectively. Automatically delineated regions yielded values of 0.29 and 0.19 μmol/cm^3^ per minute for gray matter and white matter, respectively, averaged over nine patients with a CoV of less than 15%. However, especially the white matter estimate based on the latter method was contaminated with some gray matter spill in.

Previously, Heiss and co-workers have acquired CMR_glu_ data on an HRRT scanner [15]. Using fixed rate constants from the literature, they found similar gray and white matter CMR_glu_ values with a GM/WM ratio of 2.7, which is only slightly lower than the present ratio for manually drawn regions. Therefore, it seems that a static scanning protocol, together with fixed rate constants, is a valid approximation of full kinetic modeling in the case of healthy volunteers. Nevertheless, it should be noted that the assumption of fixed rate constants may not be valid in certain clinically relevant patient populations, such as those having diabetes [31], characterized by a.o. hyperglycemia and hyperinsulinemia which both affect the blood vessel wall and the permeability surface area product [32], and decreased glucose metabolism [33]. Furthermore, normal fixed rate constants are not automatically applicable to flow-limited states [34].

The good correlation between Patlak and NLR results (slope 0.96, *r*^2^ 0.98) implies that this linearization is a valid approach, as shown previously [7]. The underestimation of 4% is likely due to the blood volume (5%) that is taken into account in the NLR analysis, but not in the Patlak linearization. Parametric *K*_i_ images without smoothing showed good correlation with regional *K*_i_ values (*r*^2^ of 0.99) with a slope of 1.04, probably induced by differences in noise and tissue heterogeneity present in regional- versus voxel-based TACs. Smoothing the dynamic ^18^ F]FDG images resulted in a lower slope and poorer correlation, and this is therefore not recommended.

Average CBF values of 0.43 and 0.13 mL/cm^3^ per minute were obtained for manually drawn gray and white matter regions, respectively. These values are in line with the recent data acquired on an HRRT by Walker and co-workers [17], who found values of 0.44 and 0.15 mL/cm^3^ per minute (Table 3). The good correlation between BFM and NLR results implies that this linearization is a valid approach.

We found an FDG extraction fraction of 18%, which is in line with earlier reported values of approximately 20% [35], determined using the double-indicator method [36]. To our knowledge, this has not been derived from a combined CBF and CMR_glu_ (*K*_1_) measurement before.

The use of a noninvasive input function, derived from the carotid arteries, allowed for quantitatively correct estimates of regional CMR_glu_ when Patlak linearization was used. In the case of NLR, however, the more stringent requirements placed on the input function (i.e., in addition to the area under the curve, the detailed shape of the peak is needed) resulted in good CMR_glu_ estimates for only seven out of nine subjects and incorrect (with an average slope of 0.76) estimates in the other two. Nevertheless, clinical dynamic [^18^ F]FDG studies without the need for an arterial line can be performed (on a voxel-by-voxel basis) by analyzing data using the Patlak linearization.

Unfortunately, the IDIF approach was not applicable to the analysis of CBF scans. Scaling to the manual samples yielded a similar factor as for CMR_glu_ scans, but an underestimation of the peak was observed (by a factor of 3). Although ^15^O has a higher positron energy (leading to an effective spatial resolution of 3.4 in the case of ^15^O, if it were 3.0 for ^18^ F), this is not likely to be the main reason for the underestimation of the peak. Possible explanations will probably include the implementation of scatter correction as well as the performance of the reconstruction algorithm for highly localized activity distributions, as prevailing in the blood just after the [^15^O]H_2_O bolus. Therefore, optimization of the frame definition and the use of a point spread function reconstruction may lead to a better match between BSIF and IDIF.

It is of importance to note that, although the images used were smoothed 6 mm at FWHM, the IDIF applicability cannot be automatically used for data generated at other PET scanners. This still needs a validation for every single tracer, every single scanner, and each acquisition and data analysis protocol separately. The success of the use of an IDIF depends on the intrinsic spatial resolution of the scanner, which is often lower than that of the scanner used in our study, and on the iterative reconstruction algorithm used (e.g., whether or not priors are included and whether the partial volume effect is implemented in the reconstruction algorithm), determining the signal-to-noise ratio as well as the minimum frame duration that can be applied. Furthermore, the duration of tracer injection needs to be optimized since the number of frames that can be acquired during the bolus should not be too low. The exact effect of these different options cannot be predicted and needs to be tested in the way as was described in this paper.

## Conclusions

NLR-based regional CMR_glu_ and CBF values can be obtained using a high-resolution PET brain scanner in healthy humans. Good correspondence between the nonlinear and linear models was observed for the regional data, which formed the basis for the calculation of accurate parametric images. Although no test-retest study was performed, average inter-subject variability of approximately 15% was observed, which implies a good reproducibility. For studies that would only require measurements of CMR_glu_, regional Patlak-based *K*_i_ values can be derived without arterial sampling by using a carotid-based image-derived input function.

## Abbreviations

BSIF: Blood sampler derived input function; CBF: Cerebral blood flow; CoV: Coefficient of variation; CMR_glu_: Cerebral metabolic rate of glucose utilization; E: FDG extraction fraction; [^18^ F]FDG: [^18^ F]-2-fluoro-2-deoxy-d-glucose; GM: Gray matter; HRRT: High-resolution research tomograph; IDIF: Image-derived input function; *K*_i_: Net influx rate; *K*_1_: Rate of transport from blood to brain; *k*_2_: Rate of transport from brain to blood; *k*_3_: Phosphorylation rate by hexokinase; *k*_4_: Rate of Hydrolysis by glucose-6-phosphatase; NLR: Nonlinear regression; ROI: Region of interest; TAC: Time-activity curve; *V*_T_: Volume of distribution; WM: White matter.

## Competing interests

The authors declare that they have no conflict of interest.

## Authors' contributions

MD and AAL designed the study. MCH did the data quality control and drafted the manuscript. LWvG recruited and scanned the subjects, performed the data analyses, and drafted the manuscript. MCH and AAL supervised the data analyses. NJH performed the data acquisition. HNG did all sample analyses. PS inserted the arterial lines. All authors edited the manuscript. All authors read and approved the final manuscript.

## References

[B1] Lammertsma AA Brooks DJ Frackowiak RS Beaney RP Herold S Heather JD Palmer AJ Jones T Measurement of glucose utilization with [18 F]2-fluoro-2-deoxy-D-glucose: a comparison of different analytical methods J Cereb Blood Flow Metab 1987 7 161 172 10.1038/jcbfm.1987.39 3558499

[B2] Schmidt KC Lucignani G Sokoloff L Fluorine-18-fluorodeoxyglucose PET to determine regional cerebral glucose utilization: a re-examination J Nucl Med 1996 37 394 399 8667082

[B3] Huang SC Phelps ME Hoffman EJ Sideris K Selin CJ Kuhl DE Noninvasive determination of local cerebral metabolic rate of glucose in man Am J Physiol 1980 238 E69 E82 6965568 10.1152/ajpendo.1980.238.1.E69

[B4] Phelps ME Huang SC Hoffman EJ Selin C Sokoloff L Kuhl DE Tomographic measurement of local cerebral glucose metabolic rate in humans with (F-18)2-fluoro-2-deoxy-D-glucose: validation of method Ann Neurol 1979 6 371 388 10.1002/ana.410060502 117743

[B5] Reivich M Kuhl D Wolf A Greenberg J Phelps M Ido T Casella V Fowler J Hoffman E Alavi A Som P Sokoloff L The [18 F]fluorodeoxyglucose method for the measurement of local cerebral glucose utilization in man Circ Res 1979 44 127 137 10.1161/01.RES.44.1.127 363301

[B6] Sokoloff L Reivich M Kennedy C Des Rosiers MH Patlak CS Pettigrew KD Sakurada O Shinohara M The [14C]deoxyglucose method for the measurement of local cerebral glucose utilization: theory, procedure, and normal values in the conscious and anesthetized albino rat J Neurochem 1977 28 897 916 10.1111/j.1471-4159.1977.tb10649.x 864466

[B7] Wienhard K Pawlik G Herholz K Wagner R Heiss WD Estimation of local cerebral glucose utilization by positron emission tomography of [18 F]2-fluoro-2-deoxy-D-glucose: a critical appraisal of optimization procedures J Cereb Blood Flow Metab 1985 5 115 125 10.1038/jcbfm.1985.15 3871780

[B8] Yu AS Lin HD Huang SC Phelps ME Wu HM Quantification of cerebral glucose metabolic rate in mice using 18 F-FDG and small-animal PET J Nucl Med 2009 50 966 973 10.2967/jnumed.108.060533 19443595 PMC2761751

[B9] Frackowiak R Lenzi G Jones T Heather J Quantitative measurement of regional cerebral blood flow and oxygen metabolism in man using 15O and positron emission tomography: theory, procedure, and normal values J Comput Assist Tomogr 1980 6 727 736 6971299 10.1097/00004728-198012000-00001

[B10] Herscovitch P Raichle ME Effect of tissue heterogeneity on the measurement of cerebral blood flow with the equilibrium C15O2 inhalation technique J Cereb Blood Flow Metab 1983 3 407 415 10.1038/jcbfm.1983.66 6415076

[B11] Koeppe RA Holden JE Ip WR Performance comparison of parameter estimation techniques for the quantitation of local cerebral blood flow by dynamic positron computed tomography J Cereb Blood Flow Metab 1985 5 224 234 10.1038/jcbfm.1985.29 3872874

[B12] Lammertsma AA Cunningham VJ Deiber MP Heather JD Bloomfield PM Nutt J Frackowiak RS Jones T Combination of dynamic and integral methods for generating reproducible functional CBF images J Cereb Blood Flow Metab 1990 10 675 686 10.1038/jcbfm.1990.121 2384540

[B13] Silverman DH Phelps ME Application of positron emission tomography for evaluation of metabolism and blood flow in human brain: normal development, aging, dementia, and stroke Mol Genet Metab 2001 74 128 138 10.1006/mgme.2001.3236 11592810

[B14] de Jong HW van Velden FH Kloet RW Buijs FL Boellaard R Lammertsma AA Performance evaluation of the ECAT HRRT: an LSO-LYSO double layer high resolution, high sensitivity scanner Phys Med Biol 2007 52 1505 1526 10.1088/0031-9155/52/5/019 17301468

[B15] Heiss WD Habedank B Klein JC Herholz K Wienhard K Lenox M Nutt R Metabolic rates in small brain nuclei determined by high-resolution PET J Nucl Med 2004 45 1811 1815 15534048

[B16] Borghammer P Hansen SB Eggers C Chakravarty M Vang K Aanerud J Hilker R Heiss WD Rodell A Munk OL Keator D Gjedde A Glucose metabolism in small subcortical structures in Parkinson's disease Acta Neurol Scand 2012 125 303 310 21692755 10.1111/j.1600-0404.2011.01556.x

[B17] Walker MD Feldmann M Matthews JC Anton-Rodriguez JM Wang S Koepp MJ Asselin MC Optimization of methods for quantification of rCBF using high-resolution [(15)O]H(2)O PET images Phys Med Biol 2012 57 2251 2271 10.1088/0031-9155/57/8/2251 22455998

[B18] Mourik JE van Velden FH Lubberink M Kloet RW van Berckel BN Lammertsma AA Boellaard R Image derived input functions for dynamic High Resolution Research Tomograph PET brain studies Neuroimage 2008 43 676 686 10.1016/j.neuroimage.2008.07.035 18707007

[B19] Mourik JE Lubberink M Klumpers UM Comans EF Lammertsma AA Boellaard R Partial volume corrected image derived input functions for dynamic PET brain studies: methodology and validation for [11C]flumazenil Neuroimage 2008 39 1041 1050 10.1016/j.neuroimage.2007.10.022 18042494

[B20] Zanotti-Fregonara P Chen K Liow JS Fujita M Innis RB Image-derived input function for brain PET studies: many challenges and few opportunities J Cereb Blood Flow Metab 2011 31 1986 1998 10.1038/jcbfm.2011.107 21811289 PMC3208145

[B21] Mourik JE Lubberink M Lammertsma AA Boellaard R Image derived input functions: effects of motion on tracer kinetic analyses Mol Imaging Biol 2011 13 25 31 10.1007/s11307-010-0301-5 20449669 PMC3023023

[B22] Boellaard R Van LA Van-Balen SC Hoving BG Lammertsma AA Characteristics of a new fully programmable blood sampling device for monitoring blood radioactivity during PET Eur J Nucl Med 2001 28 81 89 10.1007/s002590000405 11202456

[B23] Hong IK Chung ST Kim HK Kim YB Son YD Cho ZH Ultra fast symmetry and SIMD-based projection-backprojection (SSP) algorithm for 3-D PET image reconstruction IEEE Trans Med Imaging 2007 26 789 803 17679330 10.1109/tmi.2007.892644

[B24] van Velden FH Kloet RW van Berckel BN Lammertsma AA Boellaard R Accuracy of 3-dimensional reconstruction algorithms for the high-resolution research tomograph J Nucl Med 2009 50 72 80 19091902 10.2967/jnumed.108.052985

[B25] Cizek J Herholz K Vollmar S Schrader R Klein J Heiss WD Fast and robust registration of PET and MR images of human brain Neuroimage 2004 22 434 442 10.1016/j.neuroimage.2004.01.016 15110036

[B26] Svarer C Madsen K Hasselbalch SG Pinborg LH Haugbol S Frokjaer VG Holm S Paulson OB Knudsen GM MR-based automatic delineation of volumes of interest in human brain PET images using probability maps Neuroimage 2005 24 969 979 10.1016/j.neuroimage.2004.10.017 15670674

[B27] Loening AM Gambhir SS AMIDE: a free software tool for multimodality medical image analysis Mol Imaging 2003 2 131 137 10.1162/153535003322556877 14649056

[B28] Patlak CS Blasberg RG Fenstermacher JD Graphical evaluation of blood-to-brain transfer constants from multiple-time uptake data J Cereb Blood Flow Metab 1983 3 1 7 10.1038/jcbfm.1983.1 6822610

[B29] Reivich M Alavi A Wolf A Fowler J Russell J Arnett C MacGregor RR Shiue CY Atkins H Anand A Glucose metabolic rate kinetic model parameter determination in humans: the lumped constants and rate constants for [18 F]fluorodeoxyglucose and [11C]deoxyglucose J Cereb Blood Flow Metab 1985 5 179 192 10.1038/jcbfm.1985.24 3988820

[B30] Boellaard R Knaapen P Rijbroek A Luurtsema GJ Lammertsma AA Evaluation of basis function and linear least squares methods for generating parametric blood flow images using 15O-water and positron emission tomography Mol Imaging Biol 2005 7 273 285 10.1007/s11307-005-0007-2 16080023

[B31] Brooks DJ Gibbs JS Sharp P Herold S Turton DR Luthra SK Kohner EM Bloom SR Jones T Regional cerebral glucose transport in insulin-dependent diabetic patients studied using [11C]3-O-methyl-d-glucose and positron emission tomography J Cereb Blood Flow Metab 1986 6 240 244 10.1038/jcbfm.1986.37 3485643

[B32] Duckrow RB Glucose transfer into rat brain during acute and chronic hyperglycemia Metab Brain Dis 1988 3 201 209 10.1007/BF00999236 3221811

[B33] Ziegler D Langen KJ Herzog H Kuwert T Muhlen H Feinendegen LE Gries FA Cerebral glucose metabolism in type 1 diabetic patients Diabet Med 1994 11 205 209 10.1111/j.1464-5491.1994.tb02021.x 8200208

[B34] Wilson PD Huang SC Hawkins RA Single-scan Bayes estimation of cerebral glucose metabolic rate: comparison with non-Bayes single-scan methods using FDG PET scans in stroke J Cereb Blood Flow Metab 1988 8 418 425 10.1038/jcbfm.1988.78 3259241

[B35] Hasselbalch SG Knudsen GM Capaldo B Postiglione A Paulson OB Blood–brain barrier transport and brain metabolism of glucose during acute hyperglycemia in humans J Clin Endocrinol Metab 2001 86 1986 1990 10.1210/jc.86.5.1986 11344196

[B36] Knudsen GM Application of the double-indicator technique for measurement of blood–brain barrier permeability in humans Cerebrovasc Brain Metab Rev 1994 6 1 30 8186068

